# The Speech Clinic at St. Thomas's Hospital

**Published:** 1915-05-15

**Authors:** 


### THE SPEECH CLINIC AT ST. THOMAS'S HOSPITAL.

### The Case for General Adoption.

For many years the need of some special form of treatment for stammering children has been apparent to all who deal with hospital out-patients, and about a year ago the Treasurer and Governors of St. Thomas's Hospital decided to accept a kind offer from Miss Elsie Fogerty to start a Speech Clinic in the out-patient department. If such a clinic proved a success, it was hoped that it might act as a lever to arouse the educational authorities to institute similar classes at some of the London County Council schools. Thanks to the skill and understanding of Miss Fogerty and her assistant, Miss Wellesley-Reid, the results of the first year's work show a marked success, and the Speech Clinic has become a small but invaluable feature in the hospital's work. Working directly under Dr. Box, and as a part of his children's out-patient department, each case for the clinic is approved, in the first instance, by him, and then passes on to Miss Fogerty's classes.

At present the children attend the clinic twice a week, and it does not appear possible in a busy out-patient department to increase this, although the rapidity with which control is gained in the clinic suggests that daily treatment would give infinitely more speedy results.

The cases are classified thus: (1) Stammerers; (2) defective chest formation; (3) post-adenoid patients; (4) lisping or delayed baby-talk (lalling).

The method of instruction in the stammering section aims largely at the restoration of a sense of rhythm and co-ordinated movement, and at overcoming the hesitating action of the expiratory movements. Breathing exercises, vocal and musical exercises, combined with movement, form a great part of the treatment. A stammer always implies a nervous condition, and to overcome this an effort is made to induce the child to lie down and rest for some ten to fifteen minutes and entirely relax the muscles. The tensity of the muscular condition is very marked, and can be tested by the amount of relaxation which is obtained. These stammering children are, as it were, strung up to concert pitch, and in their eagerness *not* to stammer they entirely lose control of breathing and voice, with the resultant "gasp" and consequent stammer. Very often, too, a child will make up his mind that he cannot say a certain letter, and has to be educated to forget his fear, and think only of the meaning of what he wants to say.

Chest formation cases are dealt with by exercises for flexibility of the chest walls, and by many kinds of vocal and breathing exercises.

In post-adenoid cases the pharynx is usually small, and, as this interferes with respiration and good speech, exercises are used to develop the boundaries of the pharynx.

In cases of feeble-minded children, however, it is useless to concentrate on development until an idea of co-ordination of movement has been established.

Lalling (delayed speech) is often due to the foolish ways of the mother, who encourages the child to persist in "baby-talk" instead of checking the tendency to imperfect speech and helping the child gradually to more perfect articulation. Letters and combinations of a special letter with others are taught, and by degrees the difficulty may be overcome.

The Speech Clinic works in regular school terms, with formal admission, inspection, and dismissal of cases. During the first year fifty-one patients were admitted, of whom five were dismissed as unsuitable on account of age or general conditions. The cases were classified thus: Stammerers, 24; defects of chest formation, 11; post-adenoid cases, 4; lisping or delayed baby-talk, 7.

The treatment of the more serious cases of stammering is greatly assisted by $x$-ray examination, which makes it possible to ascertain the exact character of the respiratory movements during hesitation and thus render more accurate exercises for such movements practicable.

The following typical cases have been selected:—

J. R., age 9.—Word blind, terrible stammer. Attending "special school." Unable to read or write, almost unintelligible speech. Showed immediate signs of humour and intelligence. Now knows letters and can read easy sentences, and set up word puzzles at sight. Speaks without hesitation while in clinic, and can avoid hesitation unless much agitated. Great improvement in intelligence. Still attending.

E. Z., age 15.—Attending Brompton Hospital for heart. A very bad stammer, unable to obtain work. Very delicate, nervous boy, but highly intelligent. Almost at once grasped what was required of him and was able to control the nervous movements. Improved rapidly, got light work, and ceased attendance after the commencement of the war.

The organisation of the Speech Clinic lies in the lady almoner's department. Much depends on the good will and co-operation of the parents, who often need as much education, though in another direction, as the children themselves. At first the parents were inclined to treat the classes very lightly, and the attendance was consequently irregular. But by degrees they have learnt the real value of the Speech Clinic and many now help on the treatment at home, for greater progress is made when the parent will watch carefully and check slovenly speech.

In conclusion, it seems well to repeat the urgent need that exists for such classes to be established all over London. Letters are received almost daily from outside agencies requesting the hospital to accept such and such a child for the Speech Clinic. It will be readily understood that it is impossible to accede to such requests, as time and space are limited, and the clinic can only be used by *bona-fide* patients of the hospital, *i.e.* patients attending other departments; but the results of the first year's work show quite clearly how necessary it is that similar treatment should be forthcoming at various educational centres.

